# Prophylactic NS-21 maintains the skin moisture but does not reduce the severity of radiation dermatitis in patients with head and neck cancer: a randomized control trial

**DOI:** 10.1186/s13014-019-1302-4

**Published:** 2019-05-30

**Authors:** Hsiu-Ling Chou, Pei-Wei Shueng, Li-Jen Liao, Chen-Xiong Hsu, Deng-Yu Kuo, Wu-Chia Lo, Pei-Yu Hou, Li-Ying Wang, San-Fang Chou, Chen-Hsi Hsieh

**Affiliations:** 1 Department of Nursing, Far Eastern Memorial Hospital, New Taipei City, Taiwan; 2 School of Nursing, National Yang-Ming University, Taipei, Taiwan; 3 Department of Nursing, Oriental Institute of Technology, New Taipei City, Taiwan; 4 Division of Radiation Oncology, Department of Radiology, Far Eastern Memorial Hospital, 21 Sec 2, Nanya S Road, Banciao District, New Taipei City, 220 Taiwan; 5 Faculty of Medicine, School of Medicine, National Yang-Ming University, Taipei, Taiwan; 6 Department of Otolaryngology Head and Neck Surgery, Far Eastern Memorial Hospital, Taipei, Taiwan; 7 Department of Electrical Engineering, Yuan Ze University, Taoyuan, Taiwan; 8 Department of Biomedical Imaging and Radiological Sciences, National Yang-Ming University, Taipei, Taiwan; 9 School and Graduate Institute of Physical Therapy, College of Medicine, National Taiwan University, Taipei, Taiwan; 10 Physical Therapy Center, National Taiwan University Hospital, Taipei, Taiwan; 11 Department of Medical Research, Far Eastern Memorial Hospital, New Taipei City, Taiwan; 12 Institute of Traditional Medicine, School of Medicine, National Yang-Ming University, Taipei, Taiwan

**Keywords:** Head and neck cancer, Skin moisture, NS-21, Radiation dermatitis, Skin toxicity

## Abstract

**Background:**

To evaluate the practicality of NS-21 cream with regard to its skin-related toxicity in patients with head and neck cancer (HNC) who are undergoing concurrent chemoradiation therapy (CCRT) or radiotherapy (RT).

**Methods:**

Between July 2015 and November 2017, 30 HNC patients who underwent RT or CCRT were randomly allocated to receive either NS-21 or control treatment on their irradiated skin three times per day, starting at the initiation of RT or CCRT and ending 2 weeks after the completion of RT or until the appearance of grade 3 acute radiation dermatitis (ARD). Dermatitis was recorded weekly according to the Common Terminology Criteria for Adverse Events (CTCAE) version 4.0. Skin humidity was monitored by a digital moisture meter. The generalized estimating equation (GEE) and logit link function method were used for statistical analysis.

**Results:**

No serious adverse events were observed in either group. Itching dermatitis occurred on the right lower neck in one patient of the NS-21 group during the 3rd week of CCRT, but the severity was mild. The median skin moisture value at the time of the final treatment was significantly different between the study and control groups (30.6 vs. 27.3, *p* = 0.013). Additionally, there was an inverse relationship between skin moisture and ARD grade (*B* = -0.04, *p* = 0.005). The incidence of ARD at the time of the last treatment was not significantly different between the study and control groups (6.7% vs 26.7%, *p* = 0.165). The risk of grade 3 ARD for skin that had received an irradiation dose of 47–70 Gy was higher than that of skin that had received an irradiation dose ≤46 Gy (OR = 31.06, 95% CI =5.95–162.21, *p* < 0.001). Nevertheless, the risk of ARD was not significantly different between the groups (OR = 0.38, 95% CI = 0.08–1.74, *p* = 0.212).

**Conclusions:**

NS-21 was well tolerated and effective for the maintenance of skin moisture; however, there was no statistically significant reduction in the risk of ARD in HNC patients undergoing RT or CCRT when compared with HNC patients in the control group.

**Trial registration:**

The study was approved by the Institutional Review Board of Far Eastern Memorial Hospital (FEMH-IRB, 104048-F), Registered 1st June 2015,

## Introduction

Approximately 14–25% of head and neck cancer (HNC) patients experience severe acute radiation dermatitis (ARD) reactions [1, 2]. Considering the high incidence of radiation dermatitis in patients receiving radiation treatment with or without chemotherapy for HNC, this acute side effect negatively affects patient quality of life and patient compliance with treatments; furthermore, ARD may cause suspended or delayed treatment delivery, which also negatively impacts the oncological outcomes of this population [3].

Nevertheless, there are no recognized standard treatments that have been recommended for the prevention of ARD in HNC patients. Several topical agents, such as Aloe vera gel [4], topical cortisone cream [5], trolamine (Biafine) [6], and hyaluronic acid cream [5], have been applied to prevent skin dermatitis. Radiation Therapy Oncology Group (RTOG) trials were designed to confirm the role of reducing the skin toxicity of trolamine during adjuvant radiotherapy (RT), but the studies were unsuccessful [7]. Similarly, a hyaluronic acid-based emulsion did not reduce the incidence of ARD [8].

NS-21 (Plunkett Pharmaceuticals, Ltd., Sydney, Australia) is a natural cortisone-free cream that includes calendula [9], Aloe vera [4], allantoin, vitamin E [10], beta-glucan [11], hydrolyzed soy protein [12], grape seed oil [13, 14], zinc [15], honey [16], emu oil [17], avocado oil [18, 19], jojoba oil [20], rose hip oil [21, 22], urea [23] and so on. Separately, these ingredients are topical agents and are used to treat dermatitis, suggesting that NS-21 may have a potential ability to promote healing and prevent ARD. However, there are no clinical results to support this observation.

This study aimed to demonstrate the safety and efficacy of NS-21 as a prophylactic agent during concurrent chemoradiation therapy (CCRT) or RT for patients with HNC to prevent grade 3 or higher ARD during the treatment period.

## Materials and methods

### Patient population

All eligible patients had HNC proven by pathological biopsy and were evaluated by a multimodality treatment team. For inclusion, the patients had to be 20 to 80 years of age. Patients who were considered candidates for definitive treatment (RT ≥ 70 Gy) or for adjuvant treatment (RT ≥ 60–66 Gy) after surgical resection with or without concurrent chemotherapy were prospectively eligible. In addition to medical history recording and physical examination, laboratory data, fiber-optic endoscopic evaluation, imaging examinations (X-ray, computed tomography (CT) scans of the chest, magnetic resonance imaging (MRI) of the head and neck region), and a dental evaluation were included. Tumor staging was performed according to the tumor-node-metastasis staging system (American Joint Committee on Cancer cancer-staging manual, 7th edition).

Exclusion criteria included Eastern Cooperative Oncology Group Performance Status ≥2, preexisting skin ulceration or an open wound in the treatment area, a known allergy to NS-21, the presence of an inflammatory or connective tissue disorder of the skin, or the planned use of amifostine. Patients were also excluded for evidence of metastases (below the clavicle or distant metastases) confirmed by clinical or radiographic examination; for prior treatment with chemotherapy for any reason; or for RT to the HNC region, except for radioactive iodine therapy. Patients who had simultaneous primary malignancies or who were pregnant or lactating were also excluded. Patients with other systemic diseases that required the use of glucocorticoids or immunosuppressant agents prior to initiation of the study treatment were excluded.

### Ethical considerations

This study was approved by the Institutional Review Board of the Far Eastern Memorial Hospital (FEMH-IRB, 104048-F). The NS-21 cream was supplied freely by Chiaen Pharmaceutical Co., Ltd. The trial was started after receiving approval. Informed consent was obtained from all participating patients.

### Randomization

**Fig. 1:**
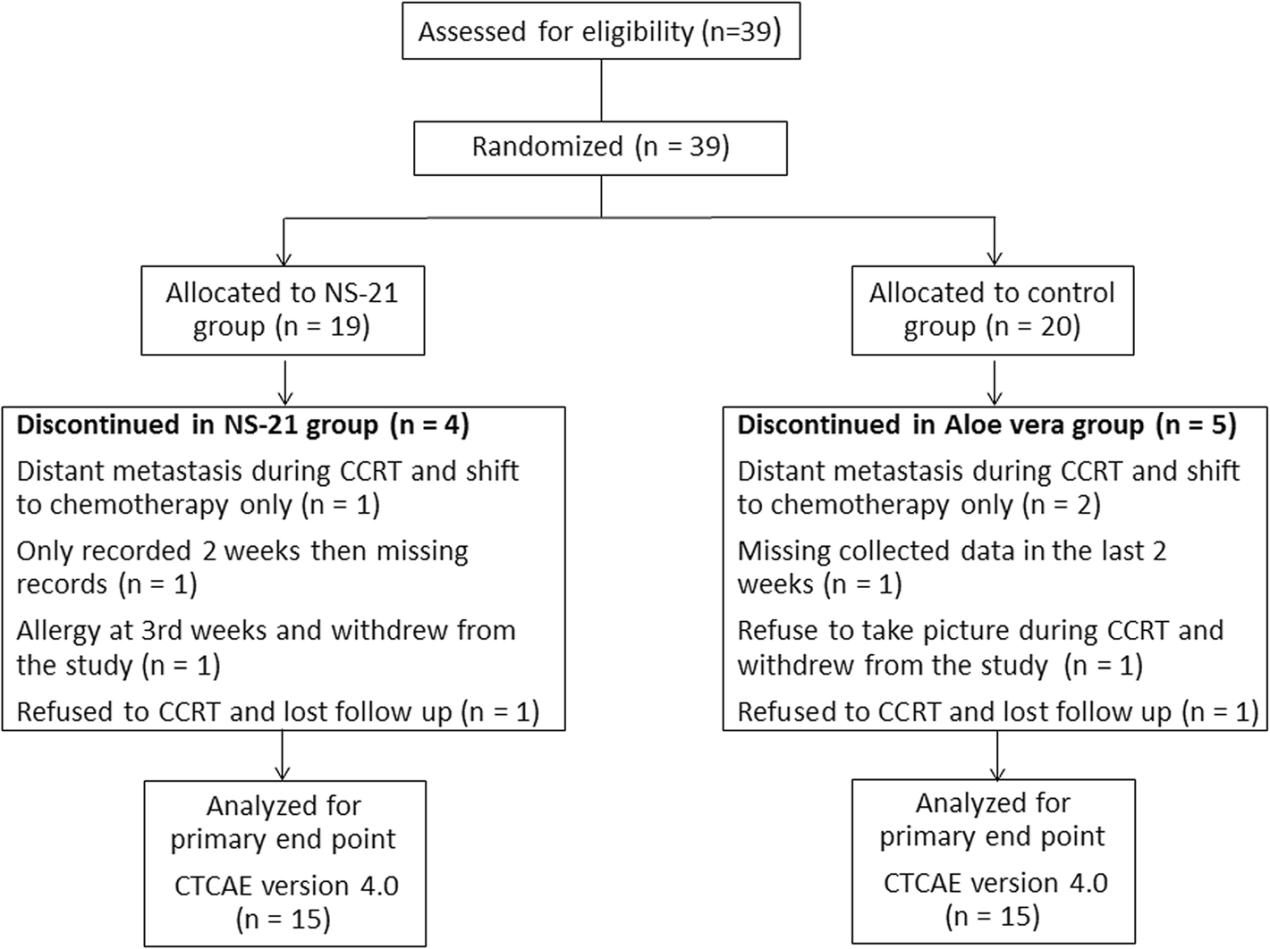
Flowchart of patients in the study. Abbreviations: CCRT, concurrent chemoradiation therapy; CTCAE, Common Terminology Criteria for Adverse Events

All patients meeting the inclusion and clearing the exclusion criteria were invited to the trial, and before the initiation of RT, the participants were randomly allocated into groups receiving NS-21 cream or a commercial Aloe vera gel. A flow diagram summarizing the processes in the present study is shown in Fig. 1. After the eligibility assessment, a randomization list was generated with a computer. A treatment allocation identification number was assigned to each enrolled patient by a random number generator. None of the study staff members were allowed access to the randomization list until the end of the study. Patients were instructed to apply NS-21 or Aloe vera gel to the irradiated area on the neck three times daily beginning on the first day of RT, continuing throughout treatments, and for 2 weeks after treatment completion. Patients were required at least 4 h between RT and the application of NS-21 or Aloe vera gel. Patients were instructed to clean the irradiated area gently and to softly pat the area dry with a cotton towel to prevent buildup of the agent on the skin and avoid an unintentional bolus effect. A bolus was permitted at the discretion of the treating physician. The NS-21 and Aloe vera gel were withdrawn immediately if an allergic reaction occurred. Upon study completion, the unused product was returned to Chiaen Pharmaceutical Co., Ltd.

#### Radiation therapy

**Fig. 2:**
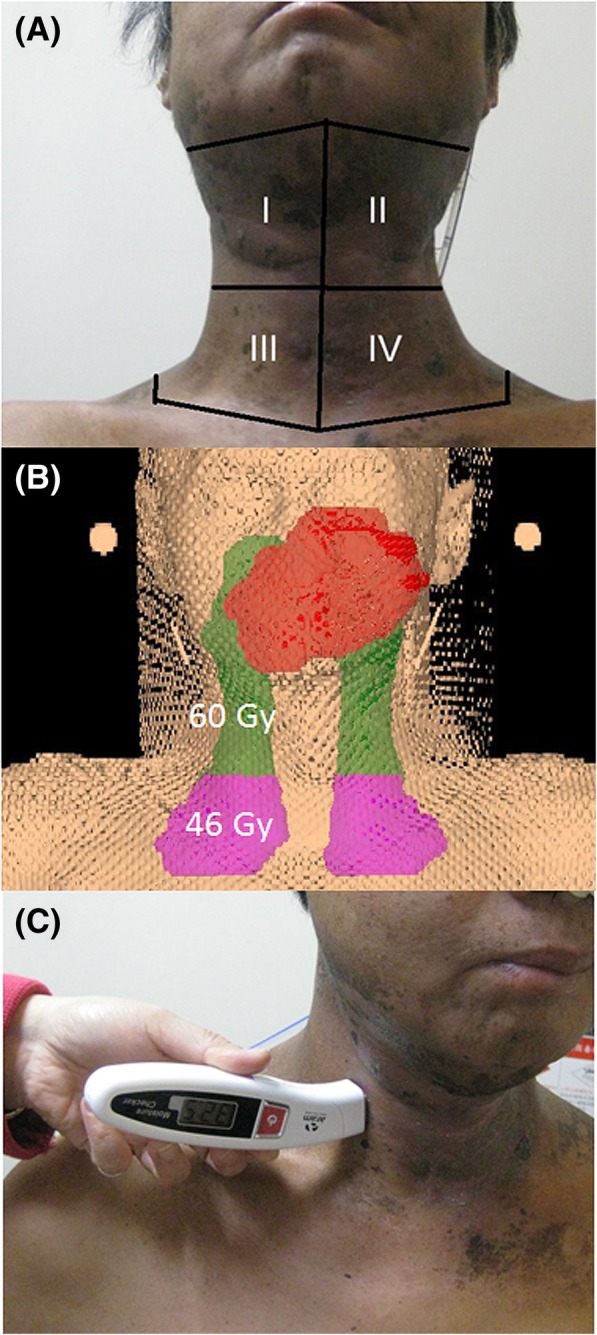
Photograph of an area of acute radiation dermatitis to be assessed and a digital moisture meter (Aramo SG, Aram HUVIS). **a** The area of acute radiation dermatitis to be recorded: (I) the right upper neck, including the lymphatic drainage area in levels I-III and Va; (II) the left upper neck, including the lymphatic drainage area in levels I-III and Va; (III) the right lower neck, including the lymphatic drainage area in level IV; and (IV) the left lower neck, including the lymphatic drainage area in level IV. **b** The accumulated doses for the upper and lower neck were 60 Gy and 46 Gy, respectively. **c** Assessment of the skin moisture value using a digital moisture meter (Aramo SG, Aram HUVIS) on the intake irradiated skin area

CT-based intensity-modulated RT (IMRT) with 6-MV photons [Helical Tomotherapy (HT), Accuray, Inc., Madison, WI, USA; Versa HD™, Elekta, Crawley, West Sussex, UK] was employed at our institution. Patients were treated with RT or CCRT to 66–70 Gy in 33–35 Gy daily fractions on five consecutive days a week with a sequential technique. The contouring system was operated using the Pinnacle 3 Treatment Planning System (Philips Healthcare, Madison, WI, USA). The clinical target volumes (CTVs) were defined as previously reported [24, 25]. Briefly, the area encompassing the gross tumor plus a 0.6- to 1-cm margin was defined as CTV1. CTV2 and CTV3 were defined as high-risk and low-risk areas of potential subclinical disease, respectively. The CTV1 and CTV2 areas plus 5 mm were used to form planning target volume (PTV)1 and PTV2, respectively, while CTV3 plus a margin of 7 mm was defined as PTV3. PTV1 received 66–70 Gy. The PTV2 dose and the PTV3 dose comprised 60 Gy in 30 fractions and 46 Gy in 23 fractions, respectively. The irradiation area on the neck was recorded as the right or left side of the ≤46 Gy area and the > 46 Gy area, respectively. (Fig. 2a, b and c).

#### Chemotherapy

Concurrent chemotherapy consisted of a weekly intravenous administration of cisplatin (30 mg/m^2^) plus fluorouracil (5-FU, 425 mg/m^2^) and leucovorin (30 mg/m^2^) [26] (Table 1).

**Table 1:** Baseline characteristics of the patients

Characteristic	Study group (*n* = 15)	Control group (*n* = 15)	*p*-value
Age (years)			
Mean ± SD	56.6 ± 10.4	56.9 ± 7.9	0.937^a^
Range	41–82	38–71	
Gender			1.000^c^
Male	14 (93.3%)	14 (93.3%)	
Female	1 (6.7%)	1 (6.7%)	
Education			0.705^b^
Less than 12 years	9 (60.0%)	10 (66.7%)	
More than 12 years	6 (40.0%)	5 (33.3%)	
Religion			0.709^b^
Buddhist	7 (46.7%)	6 (40.0%)	
Taoist	5 (33.3%)	4 (26.7%)	
Christian	3 (20.0%)	5 (33.3%)	
Marital status			0.390^c^
Without a spouse	2 (13.3%)	5 (33.3%)	
With a spouse	13 (86.7%)	10 (66.7%)	
Occupational status			0.450^c^
Homemaking or part-time employment	8 (53.3%)	11 (73.3%)	
Employed	7 (46.7%)	4 (26.7%)	
Habits related to cancer			
None	1 (6.7%)	3 (20.0%)	0.598^c^
Alcohol use	9 (60.0%)	9 (60.0%)	1.000^b^
Tobacco use	13 (86.7%)	11 (73.3%)	0.651^c^
Betel nut use	9 (60.0%)	7 (46.7%)	0.464^b^
Radiation modalities			
Versa HD™	8 (53.3%)	9 (60.0%)	0.713^b^
Helical tomotherapy	7 (46.7%)	6 (40.0%)	
Comorbidity			
None	9 (60.0%)	9 (60.0%)	1.000^b^
Diabetes	3 (20.0%)	3 (20.0%)	1.000^c^
Hypertension	4 (26.7%)	5 (33.3%)	1.000^c^
Coronary artery disease	1 (6.7%)	0	1.000^c^
Others	1 (6.7%)	2(13.4%)	
Tumor site			
Nasopharyngeal cancer	4 (26.7%)	0	
Oral cancer	7(46.7%)	10 (66.7%)	
Oropharyngeal cancer	2 (13.3%)	3 (20.0%)	
Laryngeal cancer	0	1 (6.7%)	
Hypopharyngeal cancer	2 (13.3%)	1 (6.7%)	
AJCC(7th edition) Stage			0.185^b^
Stage I	1 (6.7%)	2 (13.3%)	
Stage II	5 (33.3%)	2 (13.3%)	
Stage III	5 (33.3%)	2 (13.3%)	
Stage IV	4 (26.7%)	9 (60.0%)	
Surgery			0.128^c^
Yes	7 (46.7%)	12 (80.0%)	
No	8 (53.3%)	3 (20.0%)	
Chemotherapy			0.651^c^
Yes	12 (80.0%)	11 (73.3%)	
No	3 (20.0%)	4 (26.7%)	
Radiation dose			0.714^a^
Mean (± SD)	66.86 (± 4.20)	66.27 (± 3.85)	
Median	70	66	
Min,	60	60	
Max	70	70	

(^a^Mann-Whitney U test, ^b^Chi-square test, ^c^Fisher exact test)

### Study outcomes

The treating physicians were all radiation oncologists who specialized in HNC treatment and were blinded to which patients were treated with the NS-21 cream and with the control. Radiation dermatitis on the irradiated neck area was assessed weekly according to the NCI Common Terminology Criteria for Adverse Events (CTCAE) version 4.0 (CTCAE v 4.0, made available on May 28, 2009, at https://www.eortc.be/services/doc/ctc/CTCAE_4.03_2010-06-14_QuickReference_5x7.pdf), and the maximum grade of radiation was recorded. The relative skin humidity was monitored by a digital moisture meter (Aramo SG, Aram HUVIS) at the intake skin area. All measurements were performed in a room with consistent conditions. The data were compared with the baseline data and those recorded at weekly intervals during RT and at 2 weeks after the completion of RT.

### Statistical methods

The study variables are presented as percentages and means ± SD. The categorical variables were compared between the study group and the control group using Pearson chi-square/Fisher’s exact tests, and continuous variables (skin moisture and age) were compared using the Mann-Whitney U test between groups. The record of skin moisture and dermatitis during RT or CCRT was compiled through repeated measurements. The data were analyzed using the generalized estimating equation (GEE) and the logit link function method. Additionally, the data were adjusted by the patients during each treatment week for each patient (time) and factors (such as dose per week, radiation dose ≤46 Gy or radiation dose 47–70 Gy and moisture) to evaluate grade 3 dermatitis and skin moisture between the two groups. A *p*-value of < 0.05 was considered to be statistically significant. All statistical analyses were performed using SPSS software (Version 20.0, IBM Corporation, Armonk, NY, USA).

## Results

### Study patients

Between July 2015 and November 2017, a total of 39 patients were enrolled. Of the 39 patients, 19 and 20 patients were randomly assigned to the NS-21 and control groups, respectively (Fig. 1). After exclusion of the data mentioned above, 30 patients completed the study and were eligible for evaluation.

### Patient characteristics

The baseline patient characteristics are summarized in Table 1. The mean age for both groups was 57 years. The percentages of patients using alcohol, tobacco and betel nut were 60, 87 and 60% in the study group and 60, 73 and 46.7% in the control group, respectively. The most common tumor site was the oral cavity for both groups. The mean radiation dose for both groups was 66 Gy. Comparing the study group vs the control group, there were 53% vs 60 and 47% vs 40% patients treated with Versa HD™ and HT, respectively (*p* = 0.713). The percentages of patients treated by CCRT were 80 and 73%, respectively (*p* = 0.651). There were 47 and 53% patients with diabetes and hypertension in the study and control groups, respectively. The baseline characteristics were equally balanced between the study and control groups (Table 1).

### Adverse events

No serious adverse events were observed in either group. Itching dermatitis occurred on the right lower neck in one patient of the NS-21 group in the 3rd week of CCRT, but the severity was mild; no itching dermatitis was noted in the control group.

### Efficacy

The median skin moisture measurement at the time of final treatment was significantly different between the study and control groups (30.6 vs. 27.3, *p* = 0.013, Table 2). The ratio of patients with ARD at the last treatment time was not significantly different between the study and control groups (6.7% vs 26.7%, *p* = 0.165). Skin moisture was reduced when the cumulative RT dose was increased, as determined by univariate analysis (*B* = -0.09, *p* < 0.001). There was an inverse relationship between skin moisture and ARD grade (*B* = -0.04, *p* = 0.005). By multivariate analysis of GEE adjusted for skin moisture and dose, the risk of grade 3 ARD for skin that had received a dose of 47–70 Gy was higher than that of skin that had received an irradiation dose ≤46 Gy (OR = 31.06, 95% CI =5.95–162.21, p < 0.001). Nevertheless, the risk of ARD was not significantly different between the groups (OR = 0.38, 95% CI = 0.08–1.74, *p* = 0.212).

**Table 2:** Comparing the differences in moisture of the skin with or without prophylactic using NS-21 before and after treatment

Characteristic	Study group (*n* = 15)	Control group (*n* = 15)	*p*-value
Moisture			
Baseline, Median (P25-P75)	33.00 (23.70–47.58)	33.20 (17.80–36.98)	0.713^a^
The last time of treatment, median (P25-P75)	30.60 (27.45–32.47)	27.28 (17.80–29.80)	0.013^a^

(^a^: Mann-Whitney U test)

Abbreviation: *P25* 25th percentile, *P75* 75th percentile

#### Discussion

The current study was designed to compare NS-21 as a prophylactic agent for HNC patients undergoing RT or CCRT and demonstrated a benefit for maintaining skin moisture. However, there was no statistically significant reduction in ARD in HNC patients undergoing RT or CCRT when compared with HNC patients in the control group. Additionally, the current study also confirmed the relationships among the radiation dose, dermatitis and moisture of the skin.

Cells in the epidermis shed continuously and are replaced by keratocytes that have been formed by mitosis in the basal layer of the epidermis [27, 28]. However, basal cells are very sensitive to radiation. Generally, free radicals produced during RT will cause DNA damage followed by the destruction of proteins, lipids, carbohydrates, and complex molecules and thereby will result in (1) a marked decrease in basal cell proliferation; (2) a cascading inflammatory response; and (3) a vascular response with increasing vasodilatation, permeability and an activated coagulation system [29, 30]. Therefore, skin erythema can be observed at approximately 15–20 days of radiation as a result of a vascular response with increasing vasodilatation, permeability and activation of the coagulation system [30]. When the cumulative dose is larger than 20 Gy, the basal layer cells are destroyed, and the functions of sweat and sebaceous glands are decreased simultaneously, resulting in dry desquamation of the skin [27]. Accumulated doses of 45 to 60 Gy may damage the dermis and cause moist desquamation. In the current study, the data confirmed that the risk of grade 3 ARD for skin that received a dose of 46–70 Gy was higher than that for skin that received an irradiation dose ≤46 Gy (*p* < 0.001).

Healthy skin also plays an important role as a moisture barrier, which is achieved by the stratum corneum [31]. Corneocyte strengthening, lipid processing, and natural moisturizing factor generation contribute to the maintenance of hydration [32]. However, a cascading inflammatory response induced by radiation damages the corneum and decreases skin hydration [29, 30]. The current data show that cumulative RT dose is a risk factor for decreasing skin moisture (B = -0.09, p < 0.001), and there was an inverse relationship between skin moisture and ARD grade (*p* = 0.007). In other words, a higher dose reduced skin moisture and increased the risk of grade 3 ARD.

**Fig. 3:**
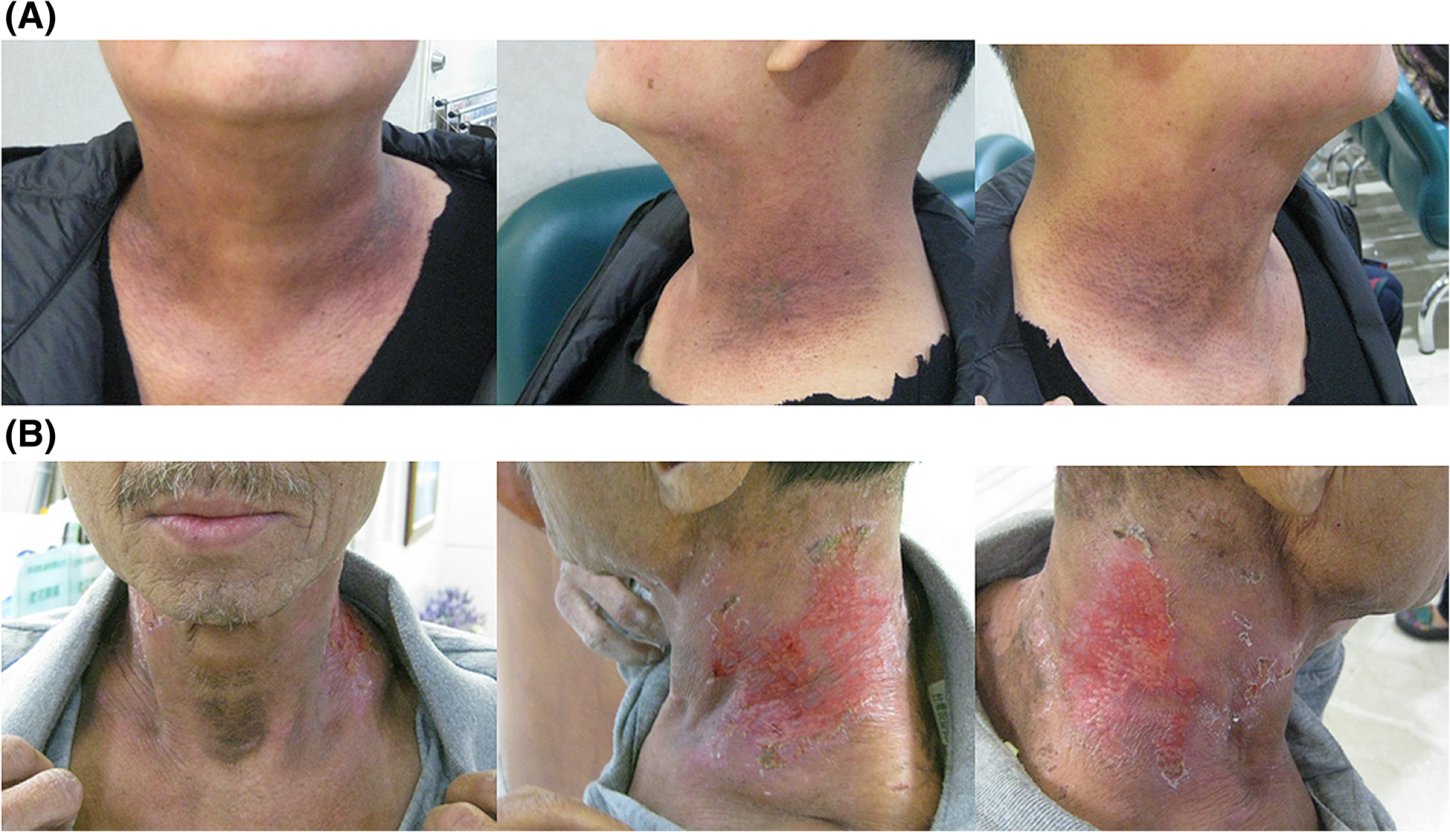
The skin condition at the beginning of prophylactic use of NS21 from the initiation of RT and to the 6th week in one representative patient from each group, showing frontal, left and right side views. **a** A 48 y/o male with nasopharyngeal cancer (cT2N2M0, stage III) who received 70 Gy in 35 fractions and who was enrolled in the study group. **b** A 60 y/o male with oropharyngeal cancer (cT2N2M0, stage IVA) who received 70 Gy in 35 fractions and who was enrolled in the control group

Compared to trolamine, calendula is 20% more effective at preventing grade 2 or higher ARD during RT for breast cancer (p < 0.001) [9]. However, a review article showed that there is no strong evidence that neither topical pharmacological interventions nor nonpharmacological topical controls can effectively prevent ARD in patients with HNC undergoing RT [33]. Another article reviewed forty-seven studies and could not provide effective suggestions for reducing ARD in HNC patients undergoing RT [34]. Tissue repair requires the involvement of the inflammatory microenvironment; however, the complicated interaction between signals produced by activated keratinocytes and the inflammatory response may also contribute to damaged skin tissue [35]. Therefore, early intervention for epidermal barrier repair may be useful in controlling dermatitis as well as in preventing its progression. In the current study, the intervention of NS-21 increased skin moisture by 12% compared to that in the control group, which may contribute to improving the integrity of skin (Fig. 3).

**Fig. 4:**
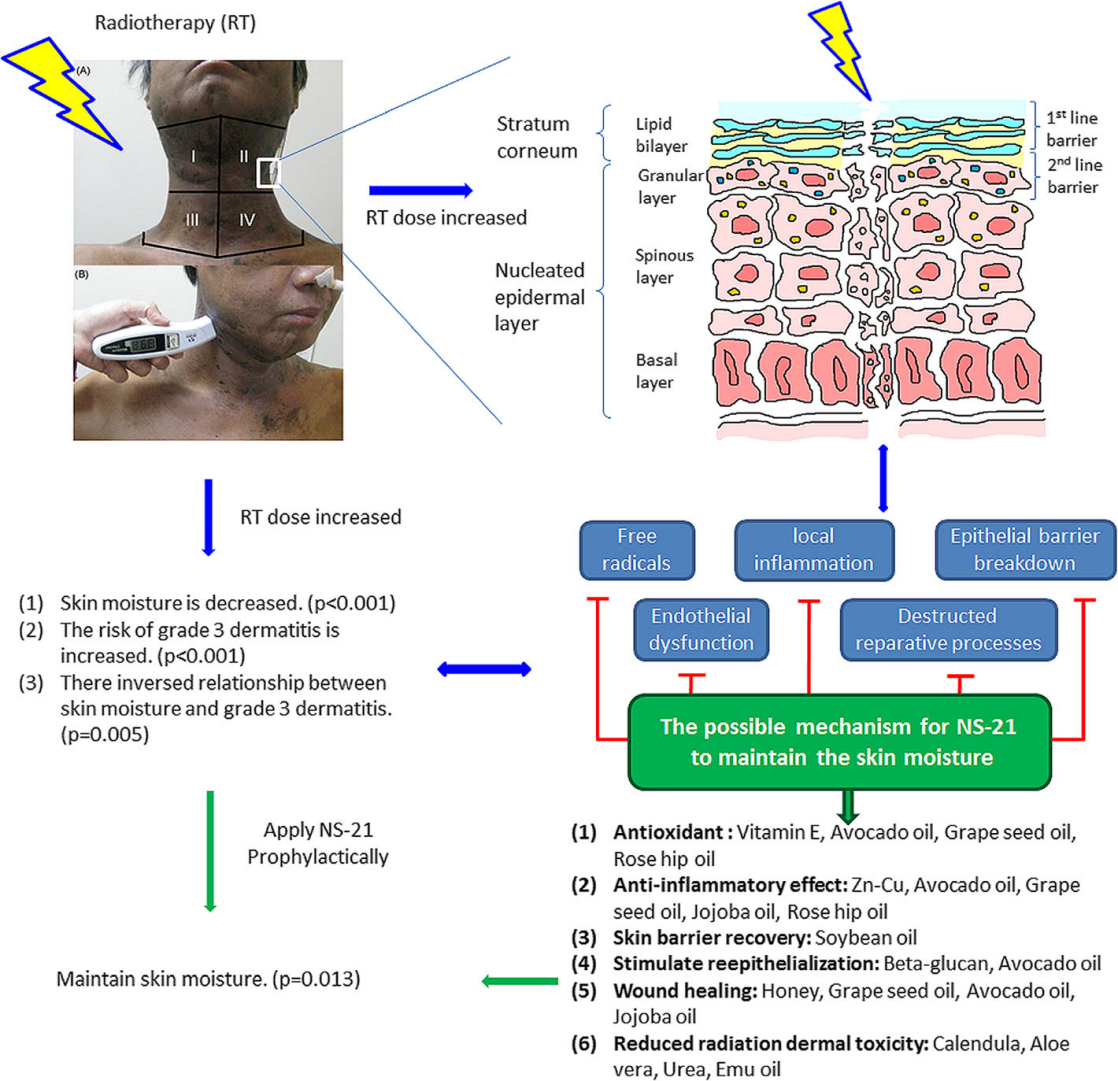
The possible mechanism by which NS-21 maintains skin moisture in patients with head and neck cancer during RT or CCRT

Interestingly, the ingredients of NS-21, such as calendula [9], beta-glucan [11], emu oil [17], urea [23] and Aloe vera-based [4], may support factors that repair the epidermal barrier (Fig. 4). For the wound healing process, the ingredients of NS-21 also include vitamin E [10], honey [16] and Zn-Cu [15]. Plant oils may play a role in promoting skin barrier homeostasis as well as antioxidative and anti-inflammatory properties to help wound healing [21], such as grape seed oil [13, 14], soybean oil [12, 36], avocado oil [19], jojoba oil [20, 37, 38] and rose hip oil [21, 22]. The results from the abovementioned studies as well as the current study suggest that NS-21 may be a potential candidate for skin moisture maintenance during RT or CCRT.

There are some limitations to the present study. First, the sample size of patients was limited, making statistical conclusions very tentative. Considering the number of cases and the use of repeated measurements in the current study, the GEE and logit link function method adjusted with clinical factors was used to analyze the skin changes and treatment results. Second, this trial lacked patient diaries to record the timing of treatment applications. We only ensured that participants applied products 3 times per day by using checklists, and we lack records of the total amount of product used. Therefore, it is difficult to confirm the relationship between timing and the amount of cream application that affected skin moisture in the current study. In the future, the use of detailed patient dairies may be warranted. Third, quality-of-life assessments were not included in the current study due to the limited number of patients. Fourth, the role of Aloe vera gel in preventing radiation dermatitis is controversial [4, 39]. The main reason that Aloe vera was used in the current study is that Aloe vera gel but not calendula has been routinely recommended to patients in our institution for several years, although the data report that calendula may decrease grade 2 or higher ARD [9]. Finally, the influences of known dermatitis risk factors, such as body mass index or body weight, are not discussed here [40].

## Conclusions

NS-21 is well tolerated and effective for the maintenance of skin moisture; however, there is no statistically significant reduction in the risk of ARD in HNC patients undergoing RT or CCRT when compared with the control group.

## Data Availability

The datasets used and/or analyzed are available from the corresponding author on reasonable request.

## Abbreviations

ARD: Acute radiation dermatitis
CI: Confidence interval.
CCRT: Concurrent chemoradiation therapy.
CT: Computed tomography.
CTCAE: Common Terminology Criteria for Adverse Events.
CTV: Clinical target volume.
GEE: Generalized estimating equation.
HNC: Head and neck cancer.
MRI: Magnetic resonance imaging.
OR: Odds ratio.
PTV: Planning target volume.
RT: Radiotherapy.
RTOG: Radiation Therapy Oncology Group.
